# Prévention de la transmission mère-enfant de l'hépatite B

**DOI:** 10.11604/pamj.2015.20.316.6193

**Published:** 2015-04-01

**Authors:** Mohamed Ould Mohamed El Agheb, Jean-Didier Grange

**Affiliations:** 1 Service d'Hépato Gastro Entérologie Hopital Tenon, 4 Rue de la Chine, 75020 Paris

**Keywords:** Hépatite virale B, grossesse, traitement antiviral, transmission mère-enfant, hepatitis B, pregnancy, antiviral treatment, mother to child transmission

## Abstract

La transmission mère-enfant du virus de l'hépatite B est la principale cause de portage chronique de l'AgHBs. La prévention de cette transmission repose principalement sur la sérovaccination du nouveau-né. En France, le dépistage prénatal de l'AgHBs est obligatoire au sixième mois de la grossesse chez toutes les femmes enceintes. Lorsque la recherche de l'AgHBs est positive chez la mère, le nouveau-né doit recevoir dès sa naissance, par voie intramusculaire et dans deux sites différents, une première injection de vaccin et une injection d'immunoglobulines anti-HBs. La vaccination est ensuite poursuivie selon le protocole recommandé. L'efficacité de la sérovaccination est supérieure à 90%, elle doit être contrôlée chez tous les enfants par un examen sérologique (AgHBs et anticorps anti-HBs) effectué à distance de la dernière injection vaccinale entre 9 et 12 mois après la naissance. Les échecs de la sérovaccination s'observent chez des femmes ayant une charge virale élevées (HBV-DNA >200 000 UI/ml). Chez ces femmes, un traitement antiviral en fin de grossesse et durant le premier mois du postpartum par un analogue nucléosidique ou nucléotidique (lamivudine, telbivudine, ou ténofovir) permet de réduire la fréquence des échecs de la sérovaccination. L'objectif était d’ étudier l'efficacité du traitement antiviral associé à la sérovaccination pour la prévention de la transmission mère-enfant du virus de l'hépatite B. Patients et méthodes: il s'agit d'une étude rétrospective incluant toutes les femmes enceintes AgHBS positif suivis en consultation dans le service d'hépato-gastroentérologie de l'Hôpital Tenon de Paris pendant une période allant de 2000 à 2013. Nous avons au cours de la période d’étude trouvé 23 femmes AgHBs positif mères de 37 enfants ont été suivies. Soixante-treize pour cent des femmes incluses étaient d'origine asiatique avec un âge moyen de 27 ans (extrêmes: 19-37 ans). Dans 81% des grossesses les femmes ont été traitées par un analogue nucléo(ti)que (lamivudine ou ténofovir). Parmi elles 68% avaient une charge virale supérieure à 5 logs au début du traitement. Quatre-vingt-dix-sept pour cent des enfants ont reçu une sérovaccination à la naissance mais seulement 86% d'entre eux avaient un schéma vaccinal complet. Soixante-treize pour cent des enfants ont eu à l’âge de 7 à 12 mois une sérologie de contrôle de la transmission (AgHBs) et de l'efficacité vaccinale (AC anti-HBs). Dans 96% l'immunité obtenue était satisfaisante avec un taux protecteur d'AC anti-HBs. le traitement par la lamivudine ou le ténofovir des femmes porteuses d'une hépatite B chronique avec une charge virale B élevée au 3^ème^ trimestre associé à la sérovaccination du nouveau-né dès la naissance permet une prévention efficace de la transmission verticale du VHB.

## Introduction

La transmission mère-enfant du virus de l'hépatite B est la principale cause de portage chronique de l'AgHBs. La prévention de cette transmission repose principalement sur la sérovaccination du nouveau-né. En France, le dépistage prénatal de l'AgHBs est obligatoire au sixième mois de la grossesse chez toutes les femmes enceintes. Lorsque la recherche de l'AgHBs est positive chez la mère, le nouveau-né doit recevoir dès sa naissance, par voie intramusculaire et dans deux sites différents, une première injection de vaccin et une injection d'immunoglobulines anti-HBs puis un rappel vaccinal à M1 et à M6. L'efficacité de la sérovaccination est supérieure à 90% [1] elle doit être contrôlée chez tous les enfants par un examen sérologique (AgHBs et anticorps anti-HBs) effectué à distance de la dernière injection vaccinale. Cependant le risque de transmission néonatal persiste car il est apparu que, même lorsque bien faite, cette sérovaccination laissait persister un risque de transmission de 5 à 10% [2]. Les études plus récentes ont montré que les facteurs prédictifs du risque de transmission étaient: la présence d'une charge virale élevée chez la mère, attestée soit indirectement par la présence ou non de l'AgHBe (transmission 9% vs 0,2%), soit directement par la présence d'un DNA > 2 x 10^5^ UI. Ce risque de transmission atteint 28 à 50% pour une charge virale > 2 x 10^8^UI [3]. La présence de l'AgHBs ou du DNA viral dans le sang du cordon, témoignant d'un passage probable du virus in utero. La présence d'une durée de travail élevée, suggérant son rôle dans le passage trans placentaire du virus au moment de l'accouchement. Un traitement par analogue nucléotidique ou nucléosidique (lamivudine ou ténofovir) réduirait le risque de transmission mère enfant chez ces femmes à forte charge virale.

## Méthodes

**Patients:** Le service de gastro-entérologie de l'hôpital Tenon suit en consultation, en collaboration avec le service de gynéco-obstétrique une cohorte de 23 patientes en âge de procréations porteuses d'une hépatite B chronique. L'hépatite chronique B était diagnostiquée pour chaque patiente enceinte sur les données virologiques, positivité de l'AgHBs et de la charge virale de l'hépatite B.

**Méthodes:** Pendant la grossesse et notamment au troisième trimestre les femmes avaient une évaluation de l'infection virale B et de la fonction hépatique comprenant: un dosage de la charge virale B, un dosage des paramètres biologiques hépatiques (ASAT, ALAT, PAL, GGT, TP). Le traitement antiviral B débuté dans la majorité des cas au début du 3ème trimestre. Il consistait dans la quasi-totalité des cas à la prescription d'un analogue nucléotidique, soit le ténofovir (245 mg/jour) soit la lamivudine (100 mg/jour). Le traitement était poursuivi jusqu'au premier ou deuxième mois du post partum. Les patientes ont été surveillées tous les 3 mois durant le traitement. A chaque visite étaient évalués: l'observance du traitement, les effets secondaires cliniques, la numération formule sanguine, le bilan hépatique et la charge virale. La réponse virale était définie comme la baisse de la charge virale de l'hépatite B de plus de 2 logs. Statistiques: les résultats sont exprimés en sommes et médianes pour les données qualitatives. Les statistiques ont été réalisées à l'aide du logiciel Epi-info (CDC Atlanta).

## Résultats

**Figure 1:**
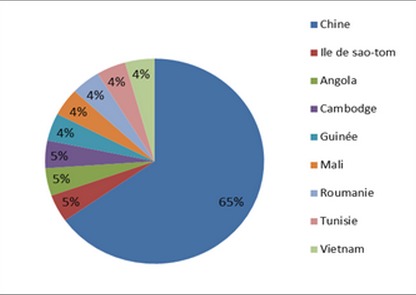
Origines géographiques des patients

**Figure 2:**
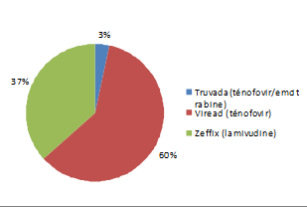
Nature du traitement antiviral

**Figure 3:**
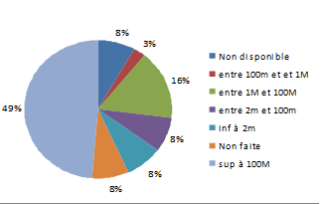
Charge virale au début du traitement (UI/ml)

**Figure 4:**
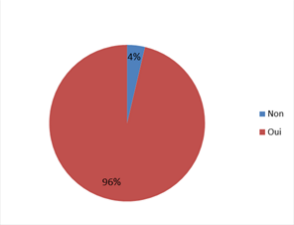
Enfants AC anti-HBs positif et AgHBs négatifs

Le nombre total des patientes enceintes avec AgHBs positif inclues dans cette étude est de 23 patientes. Dix-sept patientes d'origine asiatique (73,91%), (Figure 1). L’âge moyen: 27 ans, (extrêmes: 19 - 37 ans). Une grossesse antérieure était retrouvée chez la majorité des patientes (79%), 2 patientes avaient un antécédent de fausse couche, tandis que 2 autres avaient eu un antécédent de mort-né. Le mode de transmission était inconnu dans les 2/3 des cas; une hépatite B chronique était présente chez les parents de 5 de nos patientes, une patiente avait un antécédent de toxicomanie iv. L'hépatite chronique B était connue avant le début de la grossesse chez 16 patientes. Un seul cas de prématurité moyenne (selon l'OMS) a été retrouvé (36 SA). Durant la période d’étude il y avait 37 naissances dont 6 enfants après une césarienne, en revanche 31 enfants étaient nés par voie basse (16,2%). (Le taux de césarienne en France est de 20,2%). Le traitement antiviral a été prescrit avant le début de la grossesse dans 8 cas, poursuivit pendant toute la grossesse et interrompit à 1 ou 2 mois postpartum, Le taux de transmission du VHB était nul dans cette dernière catégorie de patiente. Dans 60% des cas le traitement antiviral prescrit était le ténofovir, (Figure 2). Ce traitement antiviral a été débuté au 3ème trimestre dans 22 cas (60%), (Tableau 1). Toutes ces patientes traitées à partir du 3ème trimestre avaient une charge virale supérieure ou égale à 7 logs, (Figure 3). Trente-six enfants ont bénéficié d'une sérovaccination à la naissance (soit 97%); dont 34 ont reçu un premier rappel vaccinal un mois après leurs naissance (soit 92%). Le schéma vaccinal complet incluant un deuxième rappel vaccinal à l’âge de 6 mois était respecté chez 32 enfants (86%). Le contrôle de la transmission (AgHBs) et de l'efficacité de la vaccination (AC anti-HBs > 10 UI/ml) était effectué chez 27 (73%) enfants dont 26 (96%) avaient une immunité satisfaisante, (Figure 4).

**Tableau 1:** Données épidémiologiques de notre cohorte

Distribution des variables	Nombre de patientes (%)
Accouchement par voie basse	34(92%)
Césarienne	3(8%)
Accouchement à terme	35(95%)
Allaitement	27(73%)
Traitement antiviral avant la grossesse	16(43%)
Traitement antiviral débuté au 3éme trimestre	30(81%)

## Discussion

La présence d'une infection par le virus de l'hépatite B (VHB) chez une femme enceinte comporte un risque important de transmission à son enfant, et les enfants contaminés en période néo-natale deviennent porteurs chroniques dans 90% des cas. En l'absence de mesure préventive, le risque de transmission verticale était de 20% si la mère est AgHBe négatif, et 80% si l'AgHBe est positif. Le dépistage de l'hépatite B est obligatoire chez toutes les femmes enceintes au 6ème mois de grossesse et une sérovaccination doit être effectuée chez tous les nouveaux nés issus de mère AgHBs positif. Cependant le risque de transmission néonatal persiste car il est apparu que, même lorsque bien faite, cette sérovaccination laissait persister un risque de transmission de 5 à 10% [2]. Les études plus récentes ont montré que les facteurs prédictifs du risque de transmission étaient: la présence d'une charge virale élevée chez la mère, attestée soit indirectement par la présence ou non de l'AgHBe (transmission 9% vs 0,2%), soit directement par la présence d'un DNA > 2 x 10^5^UI. Ce risque de transmission atteint 28 à 50% pour une charge virale > 2 x 10^8^UI [3]. La présence de l'AgHBs ou du DNA viral dans le sang du cordon, témoignant d'un passage probable du virus in utero. La présence d'une durée de travail élevée, suggérant son rôle dans le passage trans placentaire du virus au moment de l'accouchement. Un traitement par analogue nucléotidique ou nucléosidique réduirait le risque de transmission mère enfant chez ces femmes à forte charge virale. La répartition des charges virales au cours du troisième trimestre de la grossesse dans notre série est comparable à celle d'autres séries de la littérature [4]. Elle dépend principalement de l'origine géographique des femmes. Dans notre cohorte la charge virale B en fin de grossesse et début du traitement (disponible dans 84% des cas) était < à 5 logs, entre 5-7 logs et > à 7 logs respectivement chez 6 (16%), 1 (3%) et 24 (65%) POL S et al [4], avaient retrouvés respectivement 40,4%, 21% et 65% dans une cohorte de 100 cas [4]. Dans notre série, le schéma sérovaccinal était complet chez 86% des nouveaux nés. Ce taux est supérieur à celui observé dans la plupart des études nationales et européennes malgré des référentiels bien établis. On note, cependant, un taux de 90% rapporté dans une étude australienne récente ayant inclus 398 patientes [5]. Notre étude confirme les données de la littérature sur l'efficacité des analogues nucléo (ti)sidiques (ténofovir ou lamivudine) car plus de 90% des enfants contrôlés à l’âge de 7 à 12 mois (contrôle disponible dans 73% des cas) avaient un AgHBs négatif et une bonne réponse vaccinale avec un taux d'anticorps anti-HBs >10 UI/ml (100% dans une cohorte chinoise de 256 cas) [6, 7]. Dans un cas, on note que la sérovaccination associée à un traitement par le ténofovir a été efficace (absence de transmission du VHB) alors que l'enfant issu de sa première grossesse était infecté par le VHB malgré la réalisation d'une sérovaccination complète chez l'enfant, par contre sa mère n’était pas traité pendant cette grossesse. Aucun effet indésirable lié au traitement antiviral n'a été observé pendant cette étude [6–11]. De même, il n'y a pas eu de malformation congénitale. Au terme de cette étude, il apparait important que des recommandations soient rappelées (voir éditées au sein d'un manuscrit remis aux patientes infectées par le VHB).

**Recommandations:** Dépistage de l'AgHBs obligatoirement chez les femmes enceintes au 6^ème^mois de la grossesse et si positif faire le dosage de la charge virale B. Informer la mère concernant sa positivité de l'AgHBs et transmettre le résultat au gynécologue, sage-femme, au pédiatre et proposer une consultation spécialisée en hépatologie. Dépistage en urgence en salle d'accouchement (AgHBs et charge virale si positive ou hépatite déjà connu) s'il n'a pas été fait avant ou que le résultat n'est pas disponible. Dépistage du virus de l'hépatite B dans la fratrie et proposition de vaccination des personnes séronégatives pour le VHB. Informer les parents sur la nécessité de réaliser une sérovaccination complète chez l'enfant.

## Conclusion

Le traitement par la lamivudine ou le ténofovir des femmes porteuses d'une hépatite B chronique avec une charge virale B élevée au 3 ^ème^ trimestre associé à la sérovaccination du nouveau-né dès la naissance permet une prévention efficace de la transmission verticale du VHB. Ces résultats doivent être évalués au sein d'une série plus importante permettant à la fois de confirmer l'efficacité de la sérovaccination associée au traitement antiviral sur la transmission du VHB, d’évaluer la réponse vaccinale chez l'enfant et d'effectuer un suivi médical des nouveaux nés.
